# Meta-analysis of the impacts of digital information interventions on agricultural development

**DOI:** 10.1016/j.gfs.2025.100866

**Published:** 2025-06

**Authors:** Robert H. Beach, Caleb Milliken, Kirsten Franzen, Daniel Lapidus

**Affiliations:** RTI International, 3040 E. Cornwallis Road, PO Box 12194, Research Triangle Park, NC, 27709-2194, USA

**Keywords:** Digitally enabled services, Extension, Inclusive agricultural transformation, Practice adoption, Small-scale producers

## Abstract

Digital means of delivering advisory and other information services to small-scale agricultural producers are widely expected to expand the reach of extension and other information provision services while reducing costs. However, there is currently little evidence systematically quantifying these benefits. In this paper, we synthesize quantitative information on the impacts of digital information interventions, which may integrate human support, on adoption of modern farm inputs (fertilizer and improved seeds), yield, and income, covering interventions implemented between 2005 and 2019. Our starting point for the meta-analysis was the Agriculture in the Digital Age Evidence Gap Map dataset, which provided a consistent set of studies of digital interventions, though one limitation is that it excludes more recent studies published after 2021. After applying our criteria for relevance, rigor, and availability of necessary data, we used estimates from 20 studies in our analyses where fifteen were from Sub-Saharan Africa, four from India, and one from Cambodia. Mean impacts of digital information interventions on adoption of fertilizer (+23 %; 95 % CI +6 % to +40 %), yield (+6 %; +2 % to +9 %), and income (+6 %; +2 % to +9 %) are positive while effects on adoption of improved seeds were not statistically significant. Although our analysis indicates that digital farmer services have provided benefits on average, there is considerable variability in estimates across individual studies. However, there are not enough comparable quantitative observations from the literature included within our study population to reliably further disaggregate estimated impacts (e.g., by intervention, level of human assistance, geography, modality of information delivery, farmer type). Expansion of the available evidence base to facilitate quantification of these heterogeneous impacts is needed to better inform program design to maximize effectiveness.

## Highlights

• Analyzed relevant studies from the Agriculture in the Digital Age Evidence Gap Map dataset.
• Applied meta-analysis to analyze benefits of digital delivery of information to farmers.
• Digital information interventions increased fertilizer adoption, crop yields, and farmer income.
• Study data suitable for use in meta-analysis is limited and concentrated in few countries.
• Need for more studies with disaggregated data to better assess impacts of intervention characteristics.

## 1 Introduction

Agriculture is a major source of income and employment in many rural areas (World Bank, 2018), and small-scale producers contribute substantially to global food production (Ricciardi et al., 2018). However, productivity varies dramatically around the world, and a large share of small-scale farmers are very poor (Jack, 2011). Thus, improving agricultural productivity (Rapsomanikis, 2015), market information and access (Cui et al., 2018), and access to financial tools (Benami and Carter, 2021) for small-scale producers in low- and middle-income countries (LMICs) is critical for increasing food security and reducing poverty. Expanding access to agricultural information and knowledge through extension services is a key pathway for improving outcomes, but small-scale producers often have limited access to high-quality, evidence-based advice; market information; or tools to support decision-making (Fabregas et al., 2019).

The revolution in information and communications technology (ICT) has resulted in widespread farmer access to mobile phones and other means of digital delivery for agricultural extension and advisory services at low cost (Aker, 2011). Global mobile cellular subscriptions have risen from 12.0 per 100 people in 2000 to 108.0 in 2022 (104.4 per 100 people in LMICs, 61.5 for low income countries) based on data from the World Bank World Development Indicators database (https://datacatalog.worldbank.org/dataset/world-development-indicators). In response, numerous ICT-enabled products and services have been introduced in developing countries. It is widely anticipated that digital agricultural interventions will expand the reach of agricultural advice and lower the costs of delivery, providing widespread benefits to small-scale producers (Chandra and Collis, 2021; Cole and Fernando, 2016; Schroeder et al., 2021). However, though there have been large public sector and philanthropic investments in digital farmer services (e.g., mobile money, SMS-based advisory services, market price information), there is relatively limited empirical evidence quantifying the benefits provided by digital delivery and mixed findings across studies (Aker, 2011; Aker et al., 2016; Baumüller, 2018; Deichmann et al., 2016; Nakasone et al., 2014; Porciello et al., 2021; Spielman et al., 2021).

Expanding the quantitative evidence base around the effectiveness of these options can provide valuable insights into the benefits that are being provided by these services. Utilizing parameter estimates within ex ante economic modeling also has value for assessing how expanded digital intervention use contributes to poverty reduction and inclusive agricultural transformation goals that inform resource allocation. Although there have been previous studies examining the contributions of digital technologies for small-scale producers, few have attempted to quantitatively summarize the available evidence. Fabregas et al. (2019) conducted meta-analyses of the effects text messages had on farmers’ likelihood to follow advice for purchasing lime and of the effects digital agriculture programs had on yields. They found that providing information via digital technologies increased yields by 4 % and the odds of adopting lime by 22 %. However, the findings were based on results from just seven different studies in their meta-analyses.

In this study, we leverage a large systematic review of the literature (Porciello et al., 2021) to explore the contributions of digital information interventions to agricultural development. We build upon existing literature by incorporating additional studies and examining more outcomes than previously analyzed. We identify high-quality quantitative estimates of key outcomes associated with digital interventions and conduct meta-analyses of these measures. Specifically, we examine the impacts on four outcomes of interest: adoption of fertilizer, adoption of improved seeds, crop yield, and income.^1^

## 2 Methods

### 2.1 Overview of included studies

There are relatively few studies examining the effectiveness of digital interventions that also provide the requisite data to quantitatively analyze the effect of those interventions. Our starting point for the meta-analysis was the 315 studies identified in the Agriculture in the Digital Age Evidence Gap Map (EGM) dataset (Agriculture in the Digital Age, n.d.). This dataset was the result of a systematic, scoping review of more than 7000 studies published between 2000 and 2020 (Porciello et al., 2021, 2022) and provided a database of grey literature and journal articles that explicitly considered only digital interventions in agriculture, limiting the need for a separate search strategy.

Drawing from the studies in the EGM dataset, we focused our review on experimental or quasi-experimental studies of digital agricultural interventions in Africa, South Asia, and Southeast Asia. For a study to be included in our analysis, the intervention studied must have been a *digital information* intervention aimed at improving a farm outcome. Although the EGM includes only digital interventions, many of them did not meet our criteria of a digital *information* intervention. For instance, the EGM includes studies of interventions that used drones to apply fertilizer for farmers or digital *subsidies* to purchase crop inputs, neither of which would be comparable to a digital advisory or extension intervention, which involve more direct linkages of digital interventions to farmer decision-making, as is the focus of this analysis. Note that while all studies in the dataset utilized digital interventions, many of them integrated human support, which may have implications for program efficacy and cost-effectiveness.

If studies did not employ any experimental or quasi-experimental methods or control for confounding variables (e.g., they presented only a simple difference in mean yields between farming households that owned cell phones and those that did not, without controlling for other variables), we excluded them. Further, studies needed to report an *effect size* of the intervention, along with an error or measure of variance, to be incorporated into the meta-analysis.

**Fig. 1:**
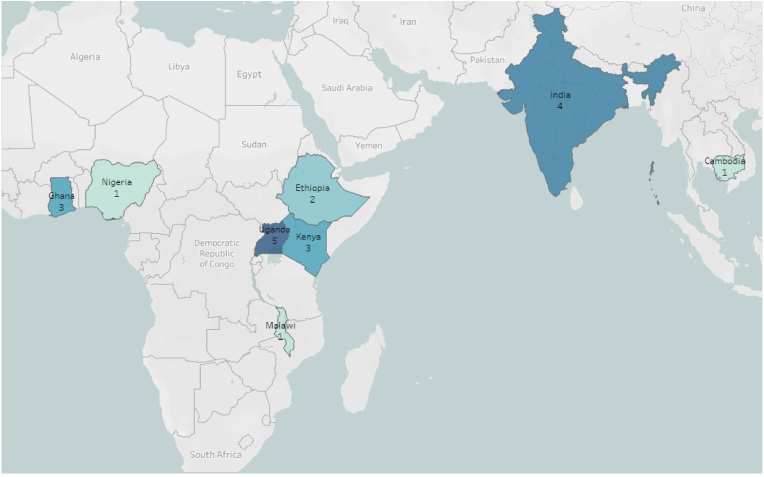
| Studies included in meta-analysis, by country. The map shows the number of studies included in the final dataset used for this study (n = 20), by country.

Of the 315 separate entries in the EGM, 52 met our initial criteria of relevance and rigor. However, upon further consideration, only 20 of those studies contained sufficient relevant data, such as effect size and standard errors, to be used in our meta-analysis. Table 1 summarizes key characteristics of those studies. These 20 studies yielded a total of 70 unique estimates, many of which reported several estimates for a single outcome (e.g., impact of the program on the yield of several separate crops). These estimates were combined to provide a single estimate of the average effect in such cases, leaving us with 35 estimates used in our meta-analyses. Those 35 values include 9 estimates of the impacts on fertilizer adoption, 4 on improved seed adoption, 13 on crop yields, and 9 on income. As shown in Fig. 1, the studies are concentrated in only 8 countries, including India, Cambodia, and 6 countries in sub-Saharan Africa, with the largest number of studies coming from Uganda (5), India (4), Kenya (3), and Ghana (3).

**Table 1:** | Summary of interventions and modalities used in each study included in this review.

Study	Intervention	Modality	Human Assisted Modality	Study Years
Arouna et al. (2021)	RiceAdvice digital application extension for rice farmers in Nigeria	Mobile Application	In-person	2016–2017
Courtois and Subervie, 2014	Mobile-based market information service for farmers in Ghana	Mobile Application	No	2009–2010
Van Campenhout et al. 2017	Community knowledge workers equipped with smartphone extension apps for maize farmers in Uganda	Mobile Application	In-person	2005–2012
Schmidt et al. (2011)	Recorded agricultural advice on a device for farmers in remote Ghana	Audio Recording	No	2009–2010
Goyal (2010)	Market information internet kiosks in farming villages for farmers in India	Kiosk	In-person	2000–2005
Sekabira and Qaim, 2017	Mobile money account for small-scale coffee farmers in Uganda	Mobile Money	No	2012–2015
Cole and Fernando (2016)	Avaaj Otalo, mobile phone–based hotline for farmers to receive advice from local extension workers	Phone Call	Remote (phone call)	2011–2013
Cole et al., 2023	Push voice calls with fertilizer recommendations in India	Phone Call	Remote (phone call)	2018–2019
Shimamoto et al. (2015)	Mobile phone call–based market information for farmers in Cambodia	Phone Call	Remote (phone call)	2012–2013
Casaburi et al. (2019)	SMS extension advice for sugar cane farmers in Kenya	SMS	No	2011–2014
Fabregas et al. (2017)	SMS 'e-extension' in Kenya	SMS	No	2014–2016
Ochieng et al. (2020)	SMS market information platform in Malawi	SMS	No	2019
Abate et al. (2019)	Video-mediated extension, Digital Green, for wheat farmers in Ethiopia	Video	In-person	2017–2018
Hörner et al. (2019)	Video-based extension for farmers in Ethiopia	Video	In-person	2016–2018
Udry et al. (2019)	Community extension agents using Android devices to show videos to farmers in northern Ghana	Video	In-person	2014–2016
Van Campenhout (2021)	Two videos, one on technical information about fertilizer use and one on return on investment of using fertilizer for rice farmers in Uganda	Video	In-person	2014
Van Campenhout et al. (2018)	ICT videos for maize farmers in Uganda	Video	In-person	2017–2018
Vandevelde et al. (2018)	Direct effects of seed selection and seed handling videos for potato farmers in Uganda	Video	In-person	2013–2014
Vasilaky et al. (2018)	Digital Green farmer training videos for rice farmers in India	Video	In-person	2014–2015
Molotsky et al. (2018)	Plantwise–Kenya, a plant health information sharing initiative, with both in-person and digital elements for farmers in Kenya	Website	In-person	2014–2017

We extracted effect sizes and other relevant information from studies that met our criteria and coded these data into an Excel spreadsheet. Some studies contained several estimates, say, for the effect of an intervention on both yield and income. Estimates were categorized into four different outcome categories: adoption of fertilizer, adoption of improved seed, yield, and income. There were many other studies that investigated the effect of interventions on a range of different outcomes; however, these outcomes were deemed outside the scope of this study, or there were too few to compile a meta-analysis. Many of the studies investigated the effect of an informational intervention on agricultural knowledge, for instance, and that was deemed outside the scope of this analysis. There were also many studies studying the effect of an intervention on the uptake of a unique agricultural practice that we did not include in the analysis (e.g., a specific post-harvest storage technique, a particular row spacing).

As estimates for yield and income were measured in many different units, currencies, and/or spatial scales, we converted all yield and income effect sizes into percent changes under the intervention relative to a measure of the control population as we felt this was the most effective and intuitive way to compare effects from a range of studies. A drawback of this method is that studies without a reported control value for the dependent variable in the absence of the intervention could not be included, as we could not calculate the percent change of an outcome under the relevant intervention. The included studies always measured adoption of improved seeds and adoption of fertilizer as an adoption rate (e.g., 38 % of study population used fertilizer), and we used the same method as we did for income and yield of measuring the percent change from the baseline/control group as our effect size.

In several instances, a single study ran and reported on multiple trials. If possible, we attempted to take the *primary* result or model specification that was preferred by the authors. However, some authors presented the results of several specifications or trials that were undertaken without providing an indication of their preferred specification. In these cases, estimates were combined into a single summary value using the methods laid out in Chapter 24 of Borenstein et al. (2009) (similar methods were also used in Fabregas et al., 2019). The single summary effect size estimate generated from combining multiple effect size estimates is calculated simply as a mean value, while the estimation of the error is adjusted to account for correlation between multiple outcomes from a single study population. This is important for multiple reasons: first, if we conducted a meta-analysis across many studies but the number of outcomes per study varied (e.g., a single study reports several specifications or outcomes for single program), then the summary effect would be assigning more weight to studies with multiple outcomes; second, treating each effect estimation from within a single study as independent likely overestimates the precision of the estimates by treating them as independent observations, while in reality they are from the same population and almost certainly correlated. It is appropriate to use these methods when multiple estimates from a single study are expected to be correlated with one another (e.g., partial or full overlap of treatment populations). We employed these methods when a single study reported multiple effects for a single outcome, like maize yield and soy yield among the same farmers.

To conduct the meta-analysis we rely on STATA's *meta* command (StataCorp, 2025). The program uses statistical methods to produce a single effect estimate from many independent effect estimates. The effect size for each study is shown in the results figures, as well as the 95 % confidence interval. The combined summary effect is a weighted average where the weights for each study are determined by how precise the estimate from that study is (i.e., weights increase as the 95 % confidence interval gets smaller). Since precision is heavily influenced by sample size, larger studies tend to have more weight in the calculation of a combined effect size.

## 3 Results

### 3.1 Impacts of digital interventions on key outcomes

#### 3.1.1 Adoption of recommended practices

There are numerous recommended practices that are expected to improve yields and/or reduce yield variability. Based on our review of available evidence, the two practices for which we had sufficient evidence to quantify impacts through a meta-analysis were the adoption of fertilizer and the adoption of improved seeds, both important practices for raising yields. There were a total of nine different studies incorporated, with all nine examining the adoption of fertilizer and four of them also assessing use of improved seeds.

**Fig. 2:**
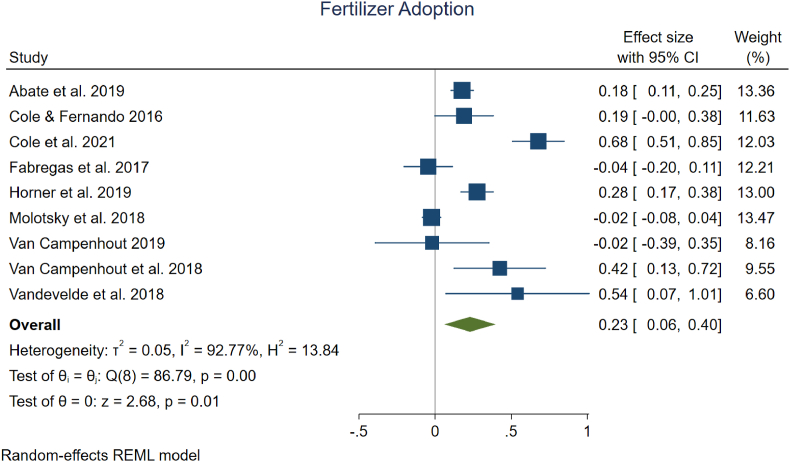
| Meta analysis on the effect of digital information interventions on the adoption of fertilizer.

Although there was variation across study findings, our meta-analysis suggests that digital interventions, on average, led to a 23 % increase in the adoption of fertilizer use among farmers (95 % confidence interval [CI], +6 % to +40 %) (Fig. 2). In addition, we present a few metrics related to heterogeneity, including τ^2^, which is an absolute measure of between-study variation in effects, I^2^, which denotes the proportion of variance in the observed effect size estimates that is due to variation in the true effect sizes, and H^2^, which describes the ratio of the observed variation to the expected variance due to sampling error. We also present homogeneity tests of effect sizes (test of θ_i_ = θ_j_) using Cochrane's Q test and a test of whether the overall effect size differs from zero. These statistics are discussed in detail in the *meta* command manual (StataCorp, 2025) and more accessibly in Borenstein et al. (2009). Overall, the evidence suggests that there is large heterogeneity in effect sizes across our studies, which is consistent with the observation that three of our studies have large mean effects between 42 % and 68 %, three have more moderate impacts between 18 % and 28 %, and the other three have small negative mean effects that fall between −2 % and −4 %.

**Fig. 3:**
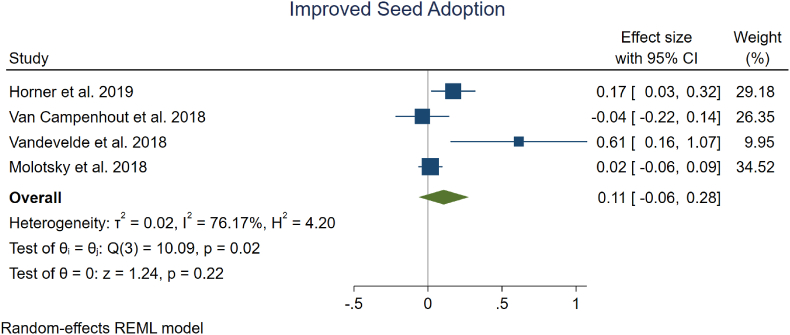
| Meta analysis on the effect of digital information interventions on the adoption of improved seeds.

There was less evidence that met the inclusion criteria for the adoption of improved seeds, only four studies. We found an average 11 % increase in improved seed usage across the four studies in Fig. 3 (95 % CI, −6 % to +28 %), but the 95 % CI includes reductions in adoption, indicating that this effect is not statistically significant.

Although adoption of fertilizer and adoption of improved seed were the two practices most commonly studied across the EGM (Porciello et al., 2021), there were many other practices that were studied for which we did not run meta-analyses due to insufficient observations (e.g., adoption of irrigation or adoption of seeding practices unique to a certain setting). Considering adoption of fertilizer and adoption of improved seed alone, our findings suggest that digital agricultural interventions in sub-Saharan Africa, India, and Cambodia have been successful in driving adoption of improved agricultural practices in a small majority of cases, though there are many programs that have negative mean effects and/or impacts that are not statistically significant.

#### 3.1.2 Impacts on yields and income

**Fig. 4:**
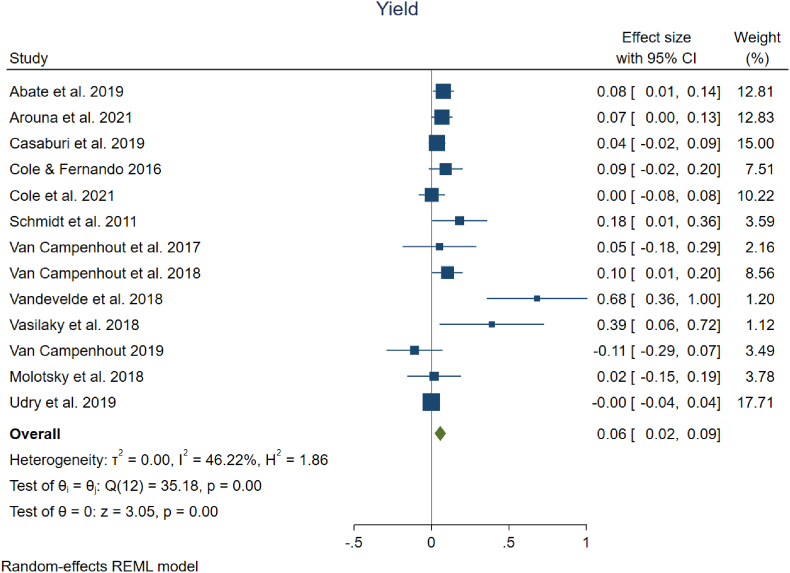
| Meta analysis on the impact of digital information interventions on farmer yield.

**Fig. 5:**
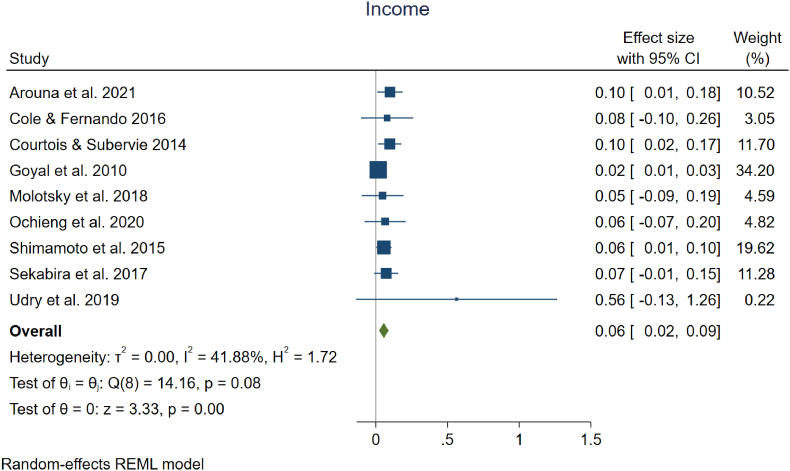
| Meta analysis on the impact of digital information interventions on farmer income and price received.

Agricultural development programs often focus on promoting the adoption of certain farming practices or technologies (such as our examples of fertilizer use and improved seeding practices), with the expectation that increased adoption will improve outcomes for the farmer (e.g., yield, income). Available studies of programs encouraging adoption do not necessarily assess the impacts on yields or income, though. Given the importance of these outcome measures for achieving inclusive agricultural transformation and poverty reduction, we considered the available evidence for the effects of digital interventions on farmer outcomes. In selecting our universe of studies with available quantitative estimates, we included all studies that had useable estimates of changes in yield and/or income measures, regardless of digital intervention (i.e., interventions were not necessarily focused on fertilizer adoption or improved seed adoption). Based on the available estimates, we found that there were indeed significant positive effects from digital interventions on small-scale producer mean yields and incomes. We estimate a 6 % average increase in yields (95 % CI, +2 % to +9 %), drawing from the 13 studies examining yield shown in Fig. 4, and a 6 % average increase in income (95 % CI, +2 % to +9 %) based on the 9 studies summarized in Fig. 5. Heterogeneity in effect sizes is moderate for yield. Two studies had negative mean impacts on yields, while over half (7 out of 13) had effects on yields that were not statistically significant. Vandevelde et al. (2018) and Vasilaky et al. (2018) had large positive mean yield gain estimates of 68 % and 39 %, respectively, but these studies also had high variance and received very low weights in calculation of the average effect. Income was the outcome measure with the least heterogeneity, with heterogeneity in effect sizes not statistically significant at the 5 % level. Mean changes in income fell between 2 % and 10 % for all but one study. That study had a mean increase in income of 56 %, though the variance around that estimate was very high and the estimate was not statistically significant. Overall, estimates from five out of the ten studies used to assess changes in income were not statistically significant.

The results for income should be interpreted carefully, due to the multiple ways in which income is measured and what was included in the analysis. Courtois et al., Goyal et al., Ochieng et al., Shimamoto et al., and Sekabira et al., all use *price received* as the dependent variable in their analyses, and those are included in the meta-analysis alongside income measures. The rationale for incorporating these studies is that interventions in these cases are typically a market information intervention where the goal is to help farmers identify more lucrative markets to increase their incomes. To the extent that additional costs are incurred to receive the higher price (e.g., additional transportation costs to reach a more distant market), using the change in price received may overstate the change in net income. Arouna et al., Cole and Fernando, Molotsky et al., and Udry et al. use income change as the dependent variable. These studies utilize slightly different measures of income but are all meant to be measures of net income (profit). Further disaggregating these findings into those from studies focusing on changes in price received versus those assessing changes in income, there is an average increase in price received of 5 % in those five studies and a 9 % increase in income reported across the four studies using a direct measure of income received.

#### 3.1.3 Comparison of digital outreach modalities

One of the key factors that may influence the outcomes achieved is the modality used to deliver the digital intervention. Table 1 summarizes the modality used to deliver information to farmers in each study included in this review as well as characterization of the human assisted modality utilized. The most common digital mode of outreach to farmers among these studies was through video extension, typically shown to farmers on tablets utilized by community representatives. Video was used in seven studies, whereas mobile applications, SMS messaging, and phone calls were used in three studies each. Additional modalities included a website, audio recording, mobile money account, and a kiosk. Although there are relatively few studies and many factors that impact intervention design, the relatively larger reliance on video for these studies may imply that intervention developers anticipate better outcomes from this modality of information delivery.

Five out of the nine studies on fertilizer adoption evaluated the impact of video-mediated extension (Abate et al., 2019; Hörner et al., 2019; Van Campenhout et al., 2018; Van Campenhout, 2021; Vandevelde et al., 2018). A meta-analysis of only those five video-mediated interventions yields a similar overall effect to the full sample, yet with more precision, with a mean of a 24 % increase in the percentage of farmers using fertilizer with a 95 % CI of +14 % to +33 %. Both of the Cole studies (2016, 2021) used telephone calls as the modality, and Fabregas et al. (2017) studied SMS extension. Molotsky et al. (2018) evaluated Plantwise–Kenya, a multifaceted program with a digital database for plant health information. Although there are not enough studies to attribute causality of adoption based on modality of the intervention with these data, the video-based extension programs have slightly more evidence of success in achieving fertilizer adoption than others. Both Cole studies show success as well, with the 2016 study using a phone hotline that farmers could call into for advice, whereas the 2021 study used push voice calls to farmers with custom fertilizer recommendations.

Among the four studies that examined adoption of improved seeds, three relied on video. Two of the three video interventions found significant and positive effects, whereas Van Campenhout et al. (2018) found results that are not significant. Molotsky et al. (2018) also found insignificant change in adoption of resistant seed varieties from the Plantwise–Kenya program's website and database. We have 13 estimates for the effects of digital interventions on yield, seven of which rely on video-mediated extension, two each on phone calls and mobile applications, and one apiece on audio recording and SMS. Another potentially important consideration is the extent to which digital information interventions rely on human assistance. Among the 20 studies included, 11 utilized in-person human assistance, 3 relied on remote human assistance via phone calls, and 6 did not use human assistance. None of the subsets of estimates broken down by modality of information delivery is significantly different from the overall average. See Supplementary data Figs. S.1–S.4 in Appendix A for a meta-analysis performed for each extension delivery modality across each outcome and Figs. S.5-S.8 for a meta-analysis disaggregated by human-assisted modality for each outcome. In the income meta-analysis, no modality is used more than twice among the nine total estimates (Fig. S.4).

Ideally, comparable intervention designs could be field-tested across several modalities to better understand which communication method is best at delivering informational messages to small-scale producers, including the importance of human assistance in leveraging digital information services. To date, there are very few studies that have included comparison of modality in their study design. One exception is Van Campenhout et al. (2018), though they were testing the incremental benefits of combining means of delivery, rather than studying the separate use of different modalities. Their experimental design included farmers that received a video only treatment, farmers that received a video + SMS reminder, and farmers that received a video + interactive voice response (IVR) to test for additional effects of these technologies. The incremental effects of SMS or IVR in addition to videos were limited, but these kind of treatment variations are valuable for generating evidence regarding effective program design.

Thus, based upon the existing literature, we have limited evidence to say anything conclusive about which *modalities* are most effective at changing farmer behavior, as the intervention design, target population, farming conditions, and a range of other factors were not comparable across studies. Thus, we cannot compare the effect of a video intervention and the effect of an SMS intervention and attribute the difference to modality. The studies of video extension services have generally been successful, however, often finding positive and statistically significant impacts on fertilizer adoption (Abate et al., 2019; Van Campenhout et al., 2018; Hörner et al., 2019; Vandevelde et al., 2018), seed adoption (Hörner et al., 2019; Vandevelde et al., 2018), and yield (Abate et al., 2019; Vandevelde et al., 2018; Vasilaky et al., 2018).

#### 3.1.4 Evidence on costs of delivery for digital extension

One of the key promises of digital extension is the theorized cost savings for providing extension to dispersed farmers around the developing world. The studies in the EGM do provide some evidence in favor of the economics of digital extension, though there are too few comparable studies to conduct a meta-analysis of benefit cost ratios (BCRs).

Even though they were not all included in the meta-analyses, several studies from the EGM quantify the cost-effectiveness of digital interventions at achieving the desired outcome (e.g., adoption of an input). Abate et al. (2019) found that, scaled to a broad population, Digital Green's (DG) video-mediated extension in Ethiopia would cost $6, $3, and $4 to achieve a single farmer adopting row planting, recommended seeding rates, and application of side dressing, respectively. Gandhi et al. (2007), also looking at DG, found it would cost around 90 % less to achieve adoption of a practice using DG compared to traditional extension. Abate et al. in Ethiopia and Vasilaky et al. in India found positive and statistically significant effects of the DG program on fertilizer adoption (Abate et al., 2019) and yield (Abate et al., 2019; Vasilaky et al., 2018). Schmidt et al. (2011), who found an 18 % increase in yields (Fig. 4) from extension messages given through audio recordings, found a BCR of 3:1 over a single season, based upon the yield increases observed. In Cole and Fernando (2016), researchers estimated that each dollar spent on the Avaaj Otalo, a mobile phone–based hotline service for farmers, returned $10 in private value.

## 4 Discussion

Although the EGM identified 315 papers providing evidence on the outcomes of digital interventions, there are relatively few that provide statistically rigorous, quantitative estimates of parameters that can be utilized in a meta-analysis. Utilizing the subset of studies that met our criteria, we found evidence of positive impacts on mean outcomes from digital interventions for three out of four outcomes studied, although there is considerable variability across the impacts estimated by individual studies. Although we used a larger dataset of studies, our results are similar to those reported by Fabregas et al. (2019). The results of our meta-analysis suggest average impacts of digital interventions on practice adoption of 23 % for fertilizer use and 11 % for improved seed use, whereas Fabregas et al. (2019) estimated the provision of digital information to increase the odds of following recommendations for lime application by 22 %. For impacts on yields, we found an average increase of 6 % compared with a 4 % increase in the Fabregas et al. study.

We feel that using these types of parameter estimates in ex ante modeling of the impacts of investments in digital interventions is valuable for assessing the potential benefits and guiding resource allocation. Such analyses provide vital information to funders that may help guide decisions regarding where to allocate resources. However, though the available evidence suggests that digital information interventions are positively impacting practice adoption, yields, and incomes, there are many remaining unanswered questions to be answered in order to better understand what is working and why. In their recent review of the literature on ICT and agricultural extension in developing countries, Spielman et al. (2021) found that “… very few studies provide evidence of increased productivity (yields) or profitability (returns) resulting from ICT use. And among those that do … questions around the rigor of causal identification … often attenuate the findings” (p. 190). Similarly, our findings are based on the small subset of the literature that provides enough quantitative information to be incorporated within a meta-analysis and may not be representative of the full set of digitally facilitated agricultural information interventions that have been implemented in LMICs. In addition, some of the available studies (e.g., Cole and Fernando, 2016) are based on programs that were implemented a decade or more ago and may be less relevant for providing current insights given major evolution in tools for digital extension. Also, there is a potential risk of publication bias tending to favor assessments that show statistically significant program impacts, which could tend to lead to overstatement of the benefits of these programs. However, we feel this risk is at least partially mitigated by incorporating evaluations from the grey literature (presumably less likely to be influenced by publication bias) in addition to journal articles. Another important consideration is that almost all of the studies that we analyze focus on one-way dissemination of information from formal extension organizations to farmers, though there are other means of introducing digital information that are being utilized (e.g., Coggins et al., 2022; Daum et al., 2022; Faxon, 2023). Interestingly, although there have been a number of programs utilizing two-way information exchange rather than just one-way transfer of information and/or that have been led by informal extension actors through means such as WhatsApp/Telegram groups (Jomantas et al., 2024; Coggins et al., 2025); farmer-led YouTube channels or Facebook groups (e.g., Faxon, 2023); and informal phone calls (Jensen, 2007; Butt, 2015), we did not identify evaluations of these types of programs within the sample used for meta-analysis. This may be important given findings that informal and/or two-way digital information interventions may be more popular with smallholder farmers (e.g., Munthali et al., 2018; Coggins et al., 2022; Lee and Suzuki, 2020).

Our results provide estimates of the overall distribution for our sample population and reveal large variation in the estimated benefits provided across studies. In addition to the differences across studies, there are substantial differences in impacts estimated within the same study. As noted earlier, in the case of studies presenting multiple values for the same outcome measure, we chose to use values from the authors’ preferred specification if available or to calculate and use an average value if not. There may be important differences in estimates for an outcome even within a study, however. For instance, a recent randomized controlled trial of video-mediated extension services found that benefits were generally concentrated among wealthier farmers (Baul et al., 2024). Ideally, one would be able to further disaggregate the available studies and assess the factors driving the observed variability. However, there are many differences in underlying study design between the available studies. Thus, the current body of evidence is insufficient to allow for definitive determination of the difference in effectiveness across interventions delivered using specific modalities in such a way that the findings could reasonably be extrapolated to similar programs applied in different settings.

Another key question that is not adequately addressed in the literature is evaluation of the incremental benefits from digital interventions relative to traditional means of extension or other information provision. Most studies do not clearly define their baseline conditions regarding utilization of traditional extension methods. Thus, though the average impacts of digital interventions are positive, it is unclear what their incremental effects are relative to traditional extension. A conservative interpretation is that the estimated impacts of digital interventions are relative to no intervention and should be compared to estimates of the effects of traditional interventions from the literature. To the extent that some of the small-scale producers in these studies were receiving traditional interventions in the baseline, the estimated impacts would be larger because they would be relative to traditional interventions rather than no intervention. One study that did explicitly compare digital delivery to traditional extension is Vasilaky et al. (2018), which found that digital delivery increased impacts by 50 % relative to traditional extension.

Even for pilots that show evidence of benefits, there remain questions regarding potential for successfully scaling up to realize the full potential of digital innovations. Abate et al. (2023) found that there has been limited progress of digital innovations in transforming agricultural markets in Africa to date. They note that many pilots have failed to scale up and argue that there is a need for more systematic assessments of digital innovations at different stages of piloting and scaling to improve our understanding of the benefits of these innovations, as well as key factors affecting successful agricultural transformation.

Also, despite the expectation that reduced costs of information delivery will be one of the largest benefits of digital interventions, there is even less evidence on cost-effectiveness than on adoption or farmer outcomes. The Agriculture in the Digital Age report finds that “… very few studies evidenced DEAS [Digitally Enabled Agricultural Services] cost-effectiveness and financial sustainability models. The lack of cost data has been highlighted in other evaluations (Laborde et al., 2020; Porciello et al., 2021, p. 40).

As noted above, we identified only five studies among those in the EGM that provided BCRs, and they examined very different interventions across multiple countries using different methodologies. This presents challenges in drawing conclusions regarding the BCR that would be expected for a specific technology and region combination. Similarly, the EGM contains studies that are highly geographically concentrated in few countries, with very little evidence from Latin America. Thus, our review is constrained to relatively few countries across sub-Saharan Africa, South Asia, and Southeast Asia, and our results should be interpreted carefully for informing those working outside of these countries. Given the substantial variation in the types of digital interventions implemented and the importance of contextual factors, there will likely always be some location- and intervention-specific variations in outcomes. Nonetheless, it is important to carefully design projects with rigorous assessment of the impacts (e.g., BCR and other measures) in mind.

In conclusion, improving the available evidence across more interventions, countries, commodities, farming systems, and other dimensions is necessary to improve our ability to assess how well alternative interventions are working, as well as the factors influencing their performance. There is a considerable need for additional rigorously designed studies collecting high-quality data that can be utilized in future meta-analyses. One area particularly in need of attention based on our review of the studies in the EGM is assessment of the cost-effectiveness of digital platforms for agricultural extension and advisory services relative to traditional means of delivery. Theoretically, the reduced costs per farmer reached is expected to be one of the key benefits of digital delivery, enabling a larger number of farmers to be reached within a given budget; however, there is surprisingly little evidence of that available in the literature. Further quantification would be valuable, as would estimation of both costs and benefits to farmers and other stakeholders associated with adopting digital interventions to improve understanding of adoption and implications for reaching inclusive agricultural transformation goals. There is a need for more regional diversity in these studies, as those included in our analyses came from only eight countries. In addition, we need to work toward more disaggregated analysis, looking at impacts that vary by type of intervention, agricultural commodity, modality of information delivery, country, and other key factors.

## CRediT authorship contribution statement

**Robert H. Beach:** Writing – review & editing, Writing – original draft, Supervision, Methodology, Conceptualization. **Caleb Milliken:** Writing – review & editing, Writing – original draft, Visualization, Methodology, Formal analysis, Data curation. **Kirsten Franzen:** Formal analysis, Data curation. **Daniel Lapidus:** Writing – review & editing, Project administration, Methodology, Conceptualization.

## Code availability

The scripts used for literature screening/selection and data analysis are available upon request.

## Funding sources

This work was supported by 10.13039/100000865Bill & Melinda Gates Foundation, Seattle, WA [contract number OPP124589].

## Declaration of competing interest

The authors declare the following financial interests/personal relationships which may be considered as potential competing interests: All authors report financial support was provided by 10.13039/100000865Bill & Melinda Gates Foundation. There are no other known competing financial interests or personal relationships that could have appeared to influence the work reported in this paper.

## Data Availability

Data will be made available on request.

## Appendix A Supplementary data

Supplementary tables (e.g., results of the meta-analysis results disaggregated by modality and by use of human assistance) are available as a separate file. Data and code are available upon request.

## Footnotes

1 Note that studies do not use identical definitions of these outcomes, but “adoption of fertilizer” refers to assessment of the proportion of farmers that are using the fertilizer recommended by the intervention (which varies across studies and may include organic (e.g., manure) and/or inorganic (e.g., urea, ammonia, di-ammonium phosphate) fertilizers) and “adoption of improved seeds” refers to assessment of the proportion of farmers that are using seed varieties that provide better quality, yield, and/or performance than the traditional seeds used in that region.
