# Supplementary material for: Meta-analysis of the impacts of digital information interventions on agricultural development
Source: Glob Food Sec. 2025 Jun;45:100866. doi: 10.1016/j.gfs.2025.100866 (PMC12167173; doi:10.1016/j.gfs.2025.100866)
Supplement: Multimedia component 1 [file mmc1.docx]

Supplementary Material


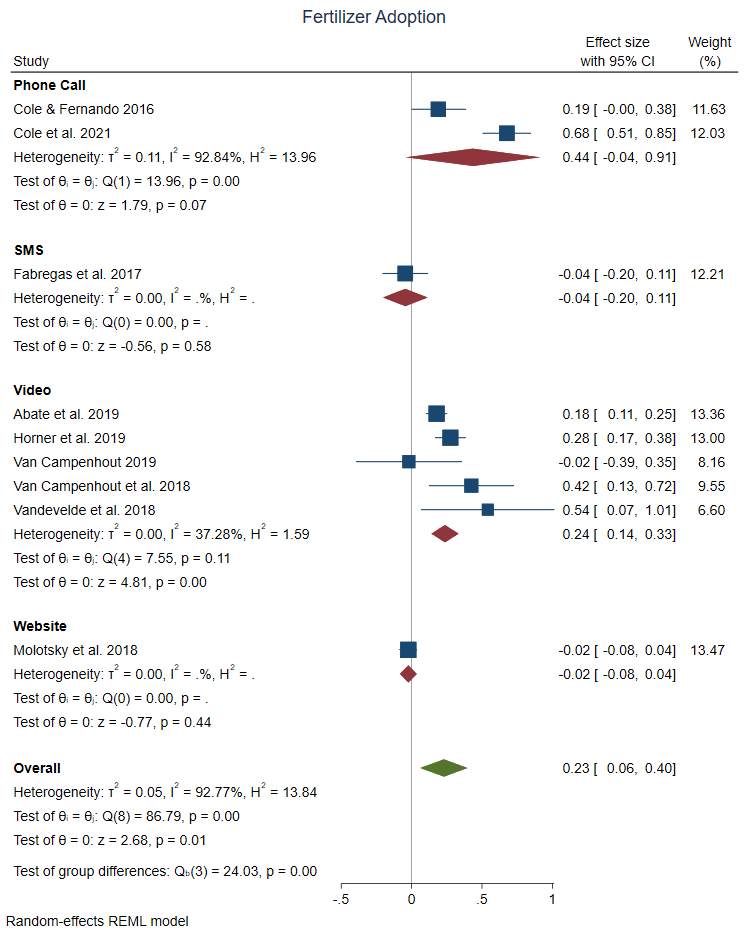


Fig. S.1 Fertilizer adoption by modality of extension


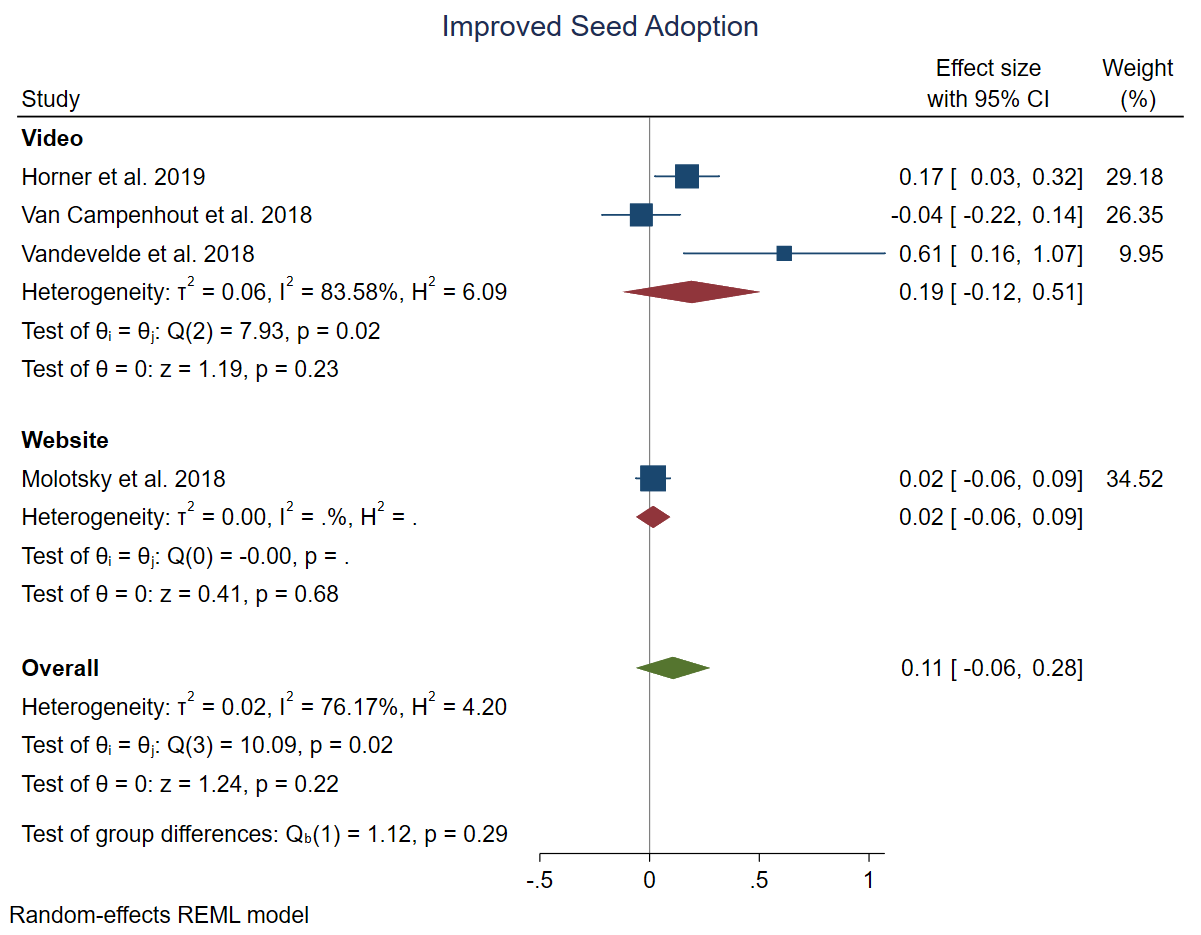


Fig. S.2 Improved seed adoption by modality of extension


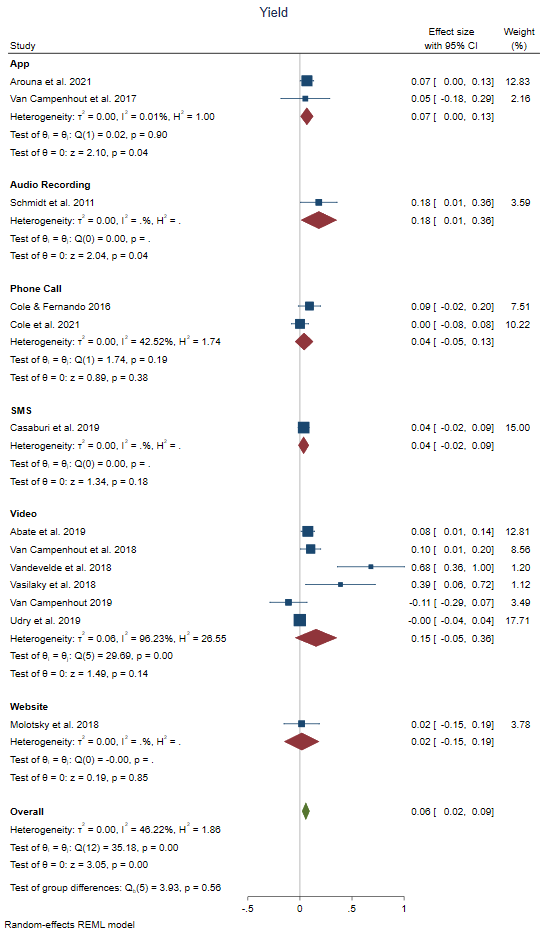


Fig. S.3. Yield change by modality of extension


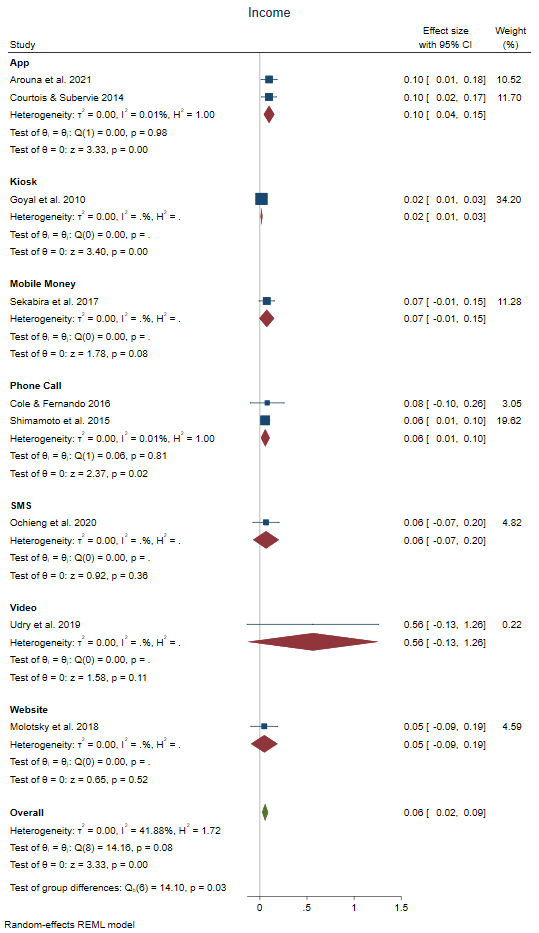


Fig. S.4 Income change by modality of extension


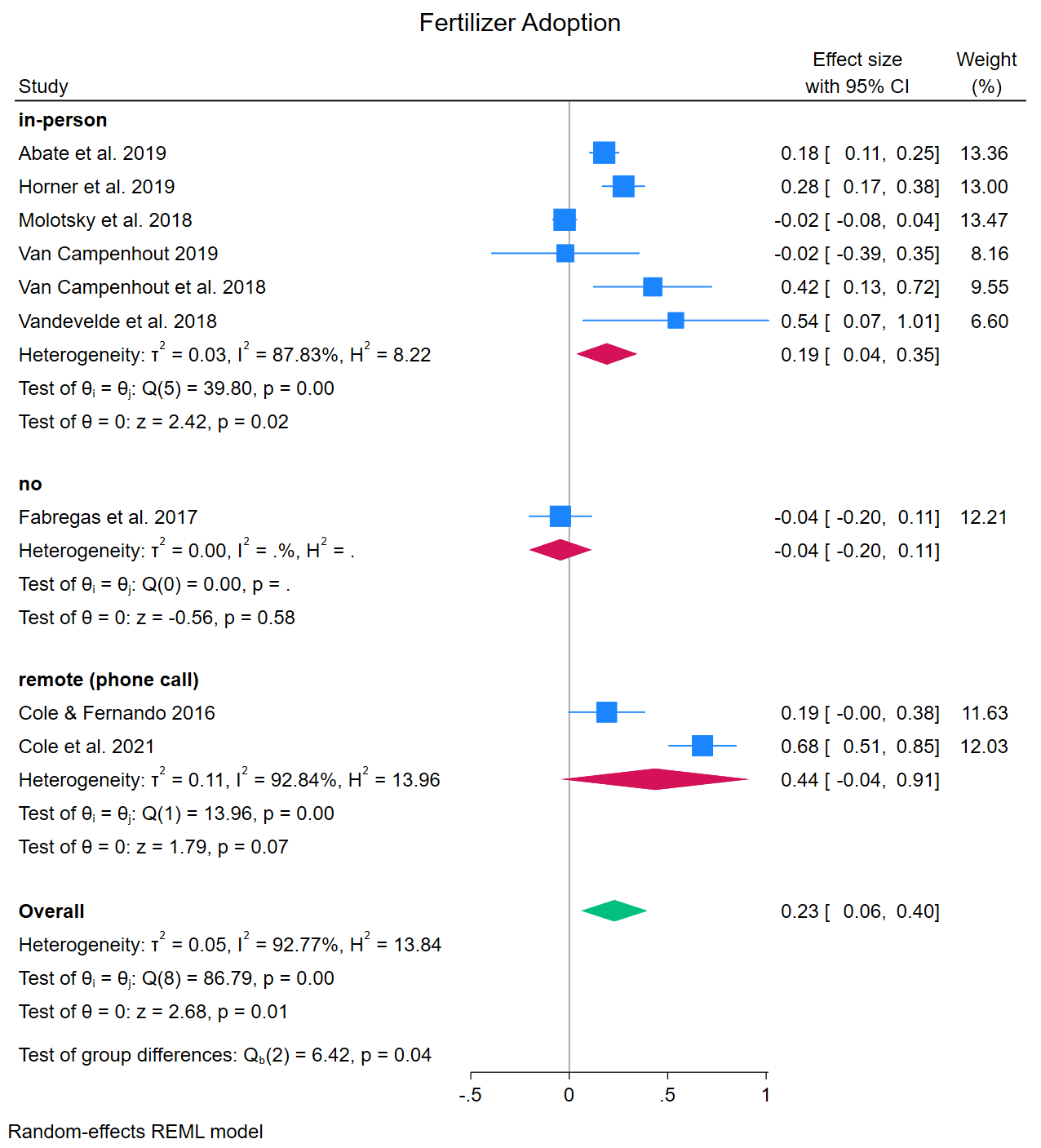


Fig. S.5. Fertilizer adoption by human-assisted modality


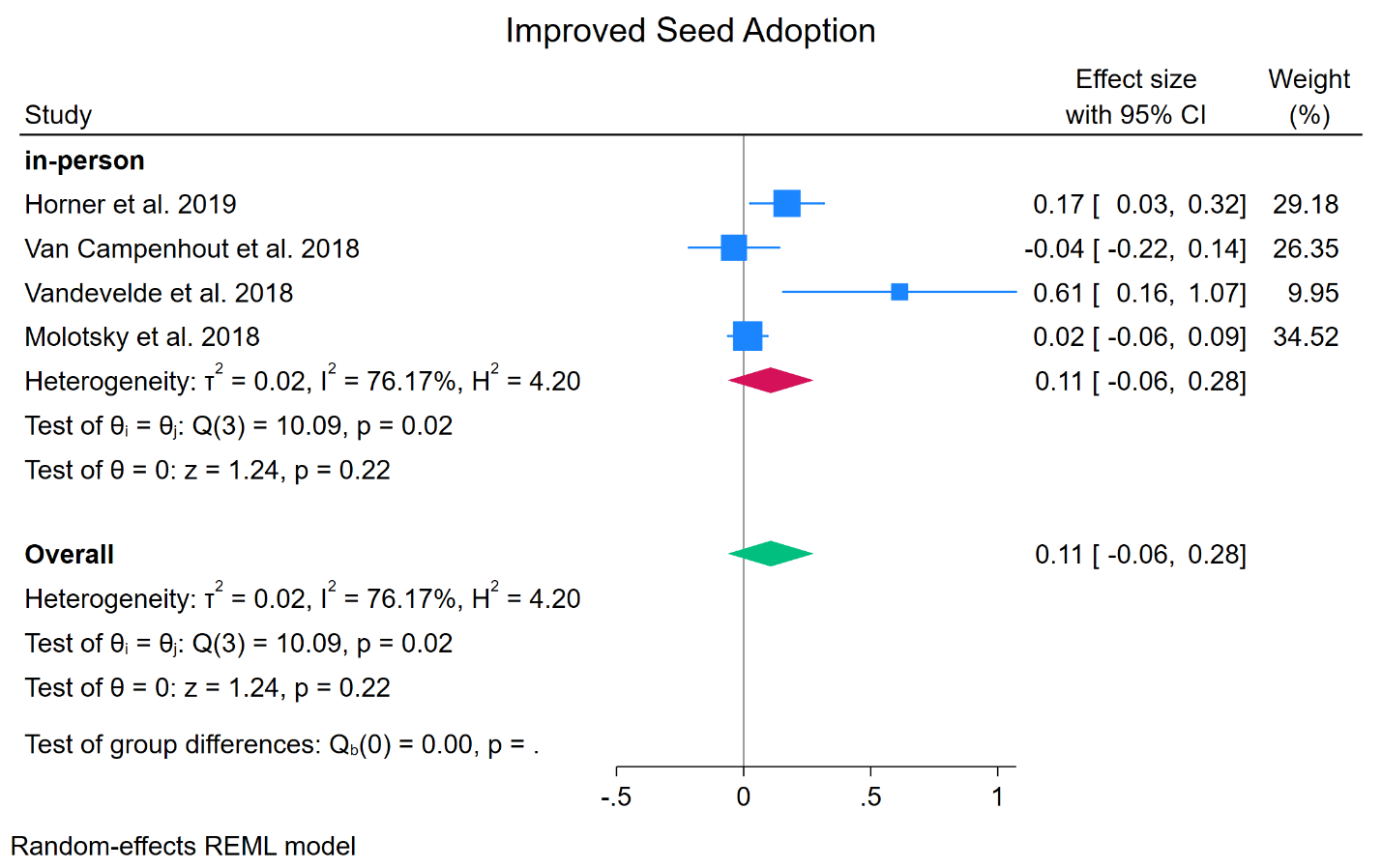
Fig. S.6. Improved seed adoption by human-assisted modality


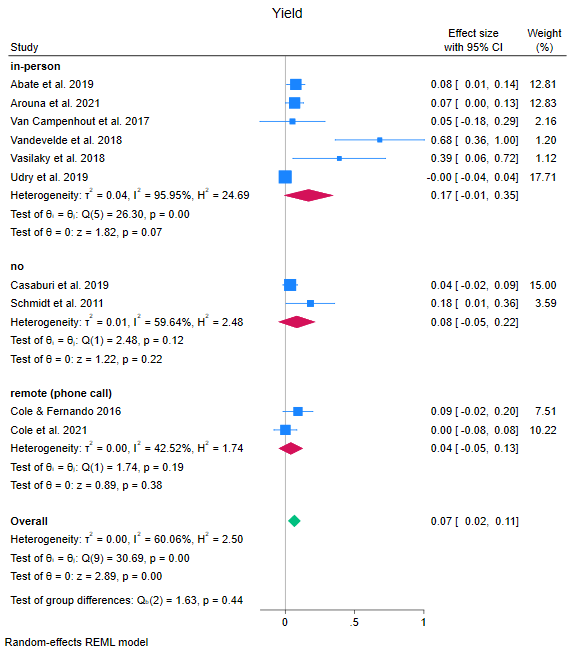


Fig. S.7. Yield change by human-assisted modality


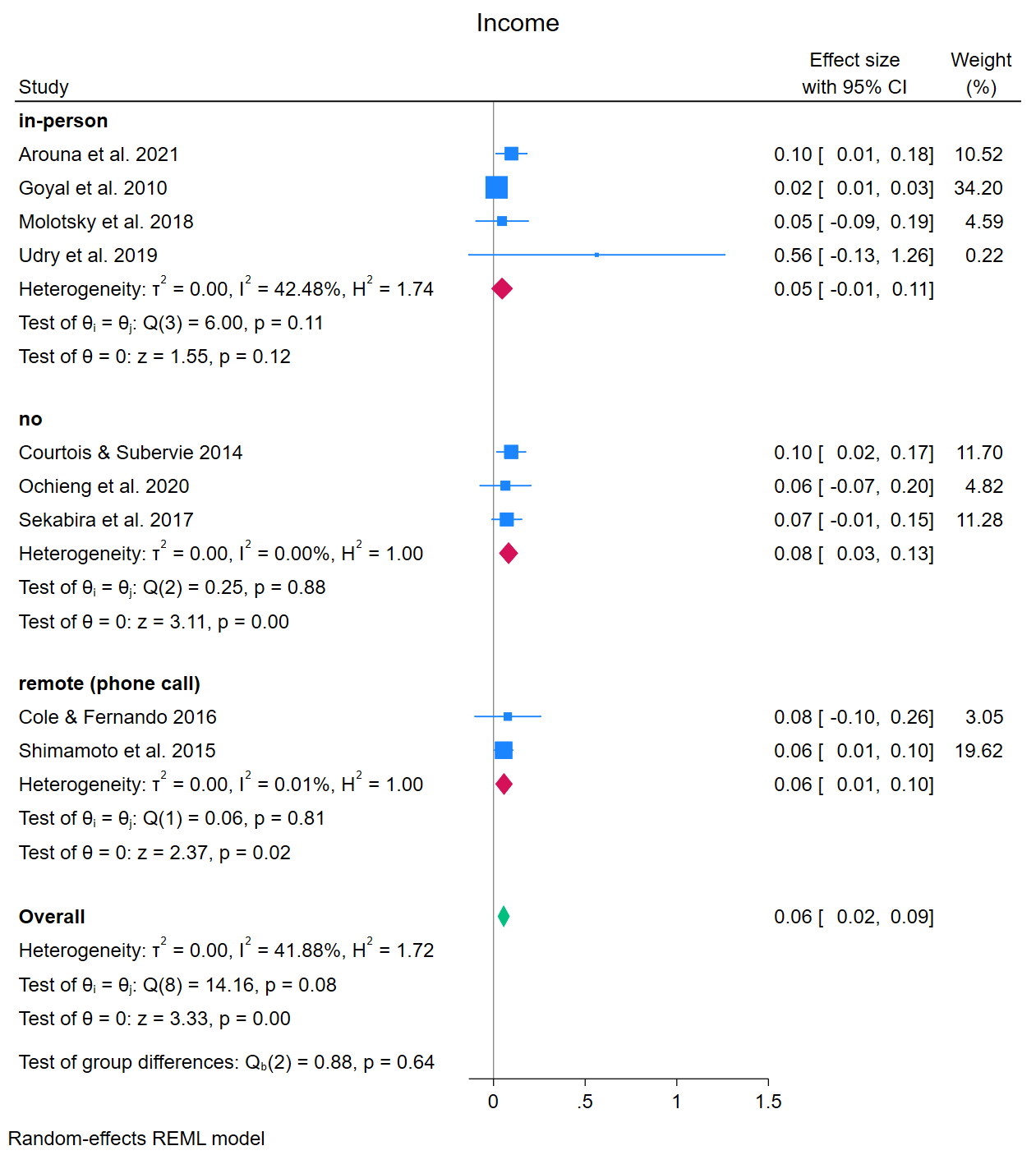


Fig. S.8. Income change by human-assisted modality
